# When School Walls Meet Emotional Hurdles: A Cross-Sectional Study on Alexithymia and School Refusal in High Schools

**DOI:** 10.5334/cie.169

**Published:** 2025-08-01

**Authors:** Mohammad Jahanaray, Ali Jahanaray, Atena Pasha

**Affiliations:** 1 University of Northern Colorado, US; 2 Clemson University, US; 3 University of South Carolina, US

**Keywords:** Alexithymia, school refusal behaviors, academic performance, emotional awareness

## Abstract

Alexithymia, the difficulty in recognizing and expressing emotions, can create significant challenges for students, contributing to anxiety and stress that predict school-refusal behaviors. This study explored how alexithymia and school refusal behaviors impact high school students’ academic performance (grade point average; GPA), considering how gender, school type, and academic major play a role. Utilizing snowball sampling, 265 students with a mean age of 16.41(*SD* = 1.7) participated in the study online, completing the Perth Alexithymia Questionnaire (PAQ) and the School Refusal Assessment Scale-Revised (SRAS-R). Through statistical analyses, including path analysis, quasi-Bayesian mediation, and Hayes moderation, we found that alexithymia and GPA were associated. Also, school refusal behaviors, like avoiding social interactions or seeking tangible rewards, did not mediate this relationship but school refusal due to avoiding negative emotions positively predicted GPA. Students in STEM (science, technology, engineering, math) fields, known for their rigorous and less emotive curricula, showed higher levels of alexithymia. In contrast, public school students were more likely to skip school for external rewards. Female students had lower alexithymia scores and higher GPAs than males. Path analysis, in turn, revealed that studying in gifted school and F2 (escaping evaluative situations) showed the largest effect sizes. School refusal findings highlight the importance of tailored interventions: public schools’ high F4 needs mentorship, peer support, and extracurriculars to counter socioeconomic refusal drivers. Also, embedding emotional literacy workshops into the curriculum, offering flexible attendance options, and fostering supportive environments with peer mentoring or teacher check-ins can counteract emotional isolation and distress, proactively addressing alexithymia’s roots and refusal triggers before they escalate.

## Introduction

*School refusal* (SR) is a broad term used to describe various types of unauthorized absences from school, including truancy, refusing to attend, difficulty remaining in school, and school withdrawal, that affect students’ social, emotional, and educational development (Fremont, 2003; Havik & Ingul, 2021; Kearney & Diliberto, 2013). This includes missing entire or partial school days, skipping classes, or unjustifiably arriving late (Kearney et al., 2010). Regular school attendance is a pivotal factor in a broad array of positive mental health, better academic outcomes, improved social and emotional behavior, and even better economic outcomes in adulthood (Kearney & Graczyk, 2020). By contrast, school refusal behaviors have been shown to negatively impact students’ academic performance (Filippello et al., 2019a).

Much research throughout the past two decades has suggested the underlying reasons for school refusal behavior. One of the most highlighted reasons is students’ unwillingness to separate from their parents and their safe zone and not wanting to experience aversive stimuli associated with their school (Mcshane et al., 2001). Other studies have noted that when school tests and assignments become too daunting for students, they may get demotivated to go to school (Willingham, 2009). Yet other studies have found that when students are at risk of violence, getting bullied, or experiencing an intimidating school environment, they avoid attendance (Kearney, 2007).

The role of parents in explaining the benefits of going to school and helping to diagnose and reduce school refusal behavior cannot be denied (Swansea, 2010) since the contextual factors of students being involved in studies about school refusal aspects cannot be neglected (Leduc et al., 2022). A few studies have examined the significant association between learning disorders (dyslexia), psychiatric disorders (attention deficit-hyperactivity disorder [ADHD], oppositional defiant disorder [ODD], or seasonal affective disorder [SAD]), and a specific learning disability (e.g., writing, reading), and their interaction with low academic achievement on school refusal or truancy (Egger et al., 2003; Filippello et al., 2019b; Wilmot et al., 2023).

Mental and psychological issues have been strongly associated with school refusal behavior or school truancy (Havik et al., 2015). For example, students who suffer from mental health problems tend to have lower school attendance, lower academic achievement and a higher chance of school dropout (Dalsgaard et al., 2020). Moreover, students with school refusal behavior refuse to go to school because they face anxiety, fear, and stress around tests, presentations, or peer interactions (Heyne et al., 2018). Research also shows that students suffering from anxiety, attachment, attention deficit and other developmental disorders, which are more prominent in female students, tend to get significantly lower grades; that is, such mental health issues, especially intellectual problems, have been shown to be strongly associated with academic achievement (Dalsgaard et al., 2020).

### Role of Alexithymia

Alexithymia is a psychological trait that causes difficulties in recognizing and describing emotions, coupled with a notable lack of imaginative fantasies, which is evident in individuals with psychosomatic disorders (Farahani et al., 2022; Sifneos, 1973). Typically, students with alexithymia become emotionally frustrated, which makes them anxious, stressed, and depressed (Akram & Arshad, 2022; Darvishi et al., 2023). Moreover, some studies also suggest that when students struggle to address emotional expression and regulation, they become vulnerable to school bullying and aggression, which are predictors of school refusal behaviors (Iuso et al., 2022; Kearney, 2007). Except for the mental and psychological factors involved with this psychological trait, some socio-demographic characteristics such as gender, education level (grade), age, major, and socioeconomic status have been associated with alexithymia (Aleisa et al., 2022).

Studies have shown that alexithymia can reduce the efficiency of the academic performance of students, especially if anxiety plays a mediating role (Romano et al., 2019). For example, some students with this trait face academic burnout, which causes them to feel emotionally exhausted and leads to lower academic performance than others (Hamaideh, 2016; Romano et al., 2019). Other factors, such as coming from a low-income family, especially for female students and medical majors, can cause a higher rate of alexithymia (Aleisa et al., 2022).

### Purpose of the Present Study

This study aimed to understand the disparities in grade point average (GPA) using alexithymia as the main explanatory variable with school refusal behaviors as a mediator to see if they can better explain the academic performance while using other moderators shown to be independently effective in both cases such as school context, major, and so on (Aleisa et al., 2022). Academic achievement/achievement in this study is referred to as GPA, as it is widely used to measure using the academic achievement index and can be interpreted as an individual’s psychological positive condition in achieving desired goals in their academics. GPA is correlated with factors such as academic self-efficacy, grade goal, and effort regulation; further, high school GPA is associated with academic performance and persistence into the second and third years at 4-year colleges and universities (Richardson et al., 2012; Westrick et al., 2015). Overall, the study aimed to answer the following two questions.

1. How does alexithymia associate with GPA among high school students, and how is this relationship mediated by school refusal behavior factors?
2. Which school type, gender, and academic major of participating students shows higher levels of alexithymia and school refusal behaviors? How do these factors moderate the relationship between GPA and alexithymia?

### Conceptual Framework

Due to the novelty of the research scope, the following conceptual framework, consisting of several theories, was chosen as appropriate. *The transactional model of stress and coping* (Lazarus & Folkman, 1984) explains why students with alexithymia do not cope well with stress and end up using maladaptive coping strategies such as school refusal, which seriously hampers academic performance. This is similar to *the theory of academic resilience* (Martin & Marsh, 2006), which advocates that counseling and the surrounding environment may aid students in overcoming emotional hurdles and minimizing the effect of alexithymia on their GPA.

**Figure 1:**
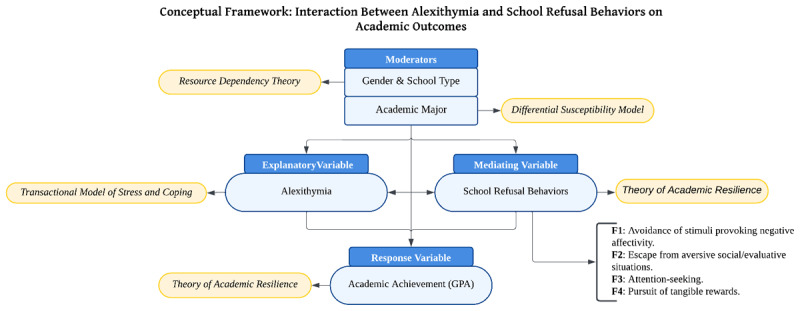
Proposed Conceptual Framework of the Study.

Further, *resource dependency theory* (Pfeffer & Salancik, 1978) presents schools with varying resource types (public, private, gifted) differing in their ability to support students with learning disorders and school refusal behaviors. Another theory explored in this study is *the theory of emotional intelligence*, which explains one’s emotions and their regulation in achieving academic success, suggesting that improving emotional intelligence may reduce school refusal and improve performance (Salovey & Mayer, 1990). Lastly, *the differential susceptibility model* (Valkenburg & Peter, 2013) relates to how one’s college major or gender creates conditions under which students respond to different educational challenges. According to them, some fields may act as a buffer or exacerbate the effects of Alexithymia and school refusal on emotional development and GPA. All the above theories form a basis for understanding emotional difficulties and interactions with behavior and academic outputs (see Figure 1).

## Methodology

### Design and Setting

This cross-sectional study utilized the snowball sampling method to investigate the relationship between alexithymia and school refusal behavior on students’ GPA while exploring the moderating effects of demographics and institutional factors and mediating effects of school refusal dimensions on GPA in Iran. Our study involved 265 high school students (106 males, 159 females) from 36 high schools with a mean age of 16.4 (*SD* = 1.7) who met the study inclusion and exclusion criteria, shown in Table 1, in 2023.

**Table 1:** Participant Inclusion and Exclusion.


INCLUSION CRITERIA	EXCLUSION

Currently enrolled in a high school	Students not currently enrolled

Aged between 14 and 18	Individuals outside the age range

Signed informed consent	Unwillingness to provide informed consent


### Instruments

The following two instruments, the Perth Alexithymia Questionnaire (PAQ) and the School Refusal Assessment Scale-Revised (SRAS-R), were used to assess alexithymia and school refusal behaviors, respectively.

#### Alexithymia

The Perth Alexithymia Questionnaire (PAQ) used in this study was designed by Preece et al. (2018) and validated by Lashkari et al. (2021) for Persian speakers. It consists of 24 self-report items that assess alexithymia. The questionnaire assesses all aspects of alexithymia, such as negative and positive emotions (Preece et al., 2018). Respondents rate the statements on a 7-point Likert scale ranging from *strongly disagree* (1) to *strongly agree* (7). Scoring followed the procedure of PAQ; that is, the total score for each subscale was calculated by summing the ratings of the items assigned to that dimension ranging from 24 to 168. The PAQ demonstrated strong reliability with a Cronbach’s alpha of 0.81, ensuring a robust assessment of alexithymia.

#### School Refusal

The primary instrument used to assess school refusal behaviors in this study was the School Refusal Assessment Scale-Revised (SRAS-R) developed by Kearney (2007) and validated by Ebrahimzadeh et al. (2020) for Persian speakers. The SRAS-R is designed to identify the underlying reasons for a child’s reluctance or refusal to attend school. In doing so, it evaluates four primary factors: avoidance of stimuli that provoke negative affectivity (F1), escape from aversive social and/or evaluative situations (F2), attention seeking (F3), and pursuit of tangible rewards (F4). The scale includes 24 items, where respondents rate their behaviors and feelings on a 7-point Likert scale ranging from *never* (0) to *always* (6). The scoring followed SRAS-S procedures as a total score for each subscale by summing the ratings of the items assigned to that factor. The SRAS-R demonstrated acceptable reliability with a Cronbach’s alpha of 0.79, showing its internal consistency in measuring school refusal behaviors.

### Data Collection Procedure

Data collection took place online in fall 2023, from August to October, using Google Forms. Upon IRB approval, informed consent forms was sent online to all participants, who were randomly selected through social media platforms such as Telegram and WhatsApp, requiring their signature before they could fill out our survey. After signing the consent form, participants answered demographic questions such as their age, school type, academic majors, and their most recent GPAs, and lastly, they filled out the scales. Researchers ensured the confidentiality and anonymity of the participants to protect their privacy. Lastly, no reciprocity was made to participants except for a certificate of appreciation for their contribution to the study.

### Data Analysis

The R programming language version 4.3.0 and SPSS (Hayes v4.3) were used for data analysis, using R packages, such as “dunn.test” and “lavaan.” The study started with descriptive analysis, demonstrating the demographics of study participants. Prior to analysis, we assessed the normality of key variables (alexithymia, F1, F2, F3, F4) using the Kolmogorov-Smirnov (with Lilliefors correction) and Shapiro-Wilk tests. Results indicated significant deviations from normality for all variables (*p* ≤ 0.005).

It is common to transform non-normal data into normal data, but we decided to keep the data as original as possible because data transformations often alter the data’s natural structure and interpretability, resulting in different substantive conclusions (Gao et al., 2008). We started with a Spearman correlation analysis to provide an overall overview of the relationships among our continuous variables. Then a series of non-parametric or robust analyses were carried out for different purposes that could handle the non-normality nature of our variables as the following provides the details:

1. In the first step, the series of univariate one-way fixed-effects Kruskal-Wallis H tests were conducted. The Kruskal-Wallis H-test for one-way analysis of variance by ranks is used to determine statistically significant differences for comparisons of three or more groups (MacFarland & Yates, 2016). We conducted separate tests for each pair of variables and used a *p*-value threshold of 0.05 to identify significant results.
2. In our second analysis, we conducted post-hoc pairwise comparisons using Dunn’s test with Bonferroni correction to identify specific group differences upon finding significant differences. We used the Bonferroni correction to control Type I errors from multiple comparisons. This adjustment divides the alpha level (usually 0.05) by the number of comparisons, maintaining the overall Type I error rate below the specified alpha level. This conservative approach increases the reliability of the results, ensuring that significant differences reflect true differences among the groups.
3. Causal mediation was explored using quasi-Bayesian confidence intervals (CI) with 1,000 simulations to assess the indirect effect of alexithymia on GPA through school refusal behaviors (F1, F2, F3, and F4) (indicated by average causal mediation effect [ACME] parameter), the direct effect of alexithymia on GPA not through the mediator (average direct effect [ADE]), and the total effect (TE) combining both pathways. The proportion of the total effect mediated was also calculated using the “mediation” package in R developed by Imai et al. (2009).
4. For moderation analysis, we used the PROCESS Macro (Hayes v4.3) in SPSS to explore the moderating effects of academic major and school type on the alexithymia-GPA relationship, leveraging its ease of testing interaction effects and its widespread use in psychological research, with bootstrapping applied to ensure robustness to non-normality. Although Macro can perform mediation, we chose the Bayesian method for its emphasis on causal mediation effects (e.g., ACME, ADE, TE), which aligns with our study’s focus on direct and indirect pathways.
5. Finally, we employed the lavaan package in R to perform path analysis to investigate the relationships among the significant relationships found in quasi-Bayesian and post-hoc analysis and their impact on academic performance as measured by GPA. Using maximum likelihood estimation with bootstrap standard errors (1,000 samples), we summarized model fit and path coefficients, including standardized estimates. However, due to path analysis’s dependence on fit indices, which necessitated excluding non-significant paths like the mediation effects of school refusal behaviors (F1–F4), we employed causal mediation analysis and the PROCESS Macro in SPSS for moderation to explore these pathways without fit constraints. This allowed us to present both significant and non-significant results comprehensively. Ultimately, we used path analysis for modeling only the significant paths to optimize model fit.

## Results

**Figure 2:**
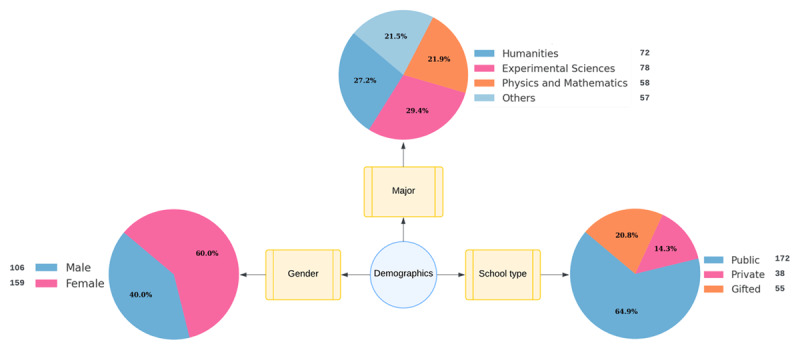
Detailed Participant Demographics.

Most of our participants were majors in experimental science, accounting for 29.4% of the sample. This figure was not markedly distinct from the representation of other academic majors. Regarding school type, students from public schools comprised 64.9% of the participant pool as seen in Table 2. The gender distribution, Figure 2 showed that females represented 60% of the sample, with males constituting the remaining 40%.

**Table 2:** Participants’ Demographic Information.


VARIABLE	CATEGORY	FREQUENCY	PERCENT

**Major**	Humanities	72	27.2

	Experimental sciences	78	29.4

	Physics and mathematics	58	21.9

	Others	57	21.5

**School**	Public	172	64.9

	Private	38	14.3

	Gifted	55	20.8

**Gender**	Male	106	40

	Female	159	60


### Spearman Correlation Analysis

The correlation analysis (Table 3) revealed key relationships among GPA, alexithymia (Alex), and other psychological factors (F1, F2, F3, F4) across 265 observations. A significant negative correlation was found between GPA and alexithymia (*r* = –0.350, *p* < 0.001), suggesting that higher levels of alexithymia correlate with lower GPA scores. Strong positive correlations were evident between F1 and F2 (*r* = 0.612, *p* < 0.001) as well as between F3 and both F1 (*r* = 0.428, *p* < 0.001) and F2 (*r* = 0.418, *p* < 0.001), indicating overlapping or interrelated constructs. Although not significantly correlated with GPA, F4 showed a modest positive correlation with F3 (*r* = 0.173, *p* = 0.005), implying some interaction among these factors.

**Table 3:** Relationship Among GPA, Alexithymia (Alex), and School Refusal (F1, F2, F3, F4).


VARIABLES	GPA	ALEX	F1	F2	F3	F4

GPA	1	–0.35**	0.03	–0.12	–0.11	–0.06

Alex	–0.35**	1	0.01	0.01	0.12*	–0.04

F1	0.033	0.01	1	0.61**	0.43**	0.09

F2	–0.17	0.01	0.61**	1	0.42**	–0.05

F3	–0.11	0.12*	0.43**	0.42**	1	0.17**

F4	–0.06	–0.04	0.09	–0.05	0.17**	1


***p < 0.001 – Very strong evidence of statistical significance. **p < 0.01 – Strong evidence of statistical significance. *p < 0.05 – Some evidence of statistical significance. *p* < 0.1 – No evidence of statistical significance.

### Kruskal-Wallis H Tests

Table 4 Kruskal-Wallis tests revealed that GPA significantly differed by gender (*p* < 0.001), major (*p* < 0.001), and school type (*p* < 0.01), indicating that academic performance may be associated with these demographic and educational factors. F2 showed significant differences by major (*p* < 0.001) among school refusal factors, suggesting that academic discipline may be related to tendencies to escape from aversive social and/or evaluative situations. Evidence also showed that the alexithymia rate was significantly different across majors (*p* < 0.05), pointing to the potential relationship between field of study and emotional processing. There was also statistical evidence supporting the fact that schools with different locale codes (public, private, and gifted) had different rates of F4 (*p* < 0.01), indicating that the type of school was important in response to school refusal behaviors and academic outcomes.

**Table 4:** Summary of Kruskal-Wallis Tests.


VARIABLE	MODERATOR	*X^2^*	*p*-VALUE

Alexithymia	Gender	0.69	0.41

F1	Gender	0.19	0.66

F2	Gender	2.24	0.14

F3	Gender	3.48	0.06

F4	Gender	0.01	0.93

GPA	Gender	27.70	<0.001***

GPA	School	10.25	0.005**

Alexithymia	Major	10.66	0.01*

F1	Major	4.84	0.184

F2	Major	8.66	0.03*

F3	Major	2.70	0.44

F4	Major	4.83	0.18

GPA	Major	58.93	<0.0001***

Alexithymia	School	3.80	0.15

F1	School	0.32	0.85

F2	School	0.20	0.90

F3	School	0.38	0.83

F4	School	10.16	0.006**


***p < 0.001 – Very strong evidence of statistical significance. **p < 0.01 – Strong evidence of statistical significance. *p < 0.05 – Some evidence of statistical significance. *p* < 0.1 – No evidence of statistical significance.

### Dunn’s Test With Bonferroni Correction (Follow-Up analysis)

Table 5 a follow-up analysis of statistically significant results on the Kruskal-Wallis tests. The differences in GPA through multiple pairwise comparisons revealed significant disparities between groups, particularly between Group 4 and all other groups (*p* < 0.001). Majors in Groups 2 and 4 showed statistically different rates of school refusal behavior in F2 (*p* < 0.05). Moreover, in analyzing F4, school types showed significant differences (*p* < 0.05): Public schools tended to have a higher rate than private schools (Group1 > Group 3). These results highlight specific group differences between GPA, F2, and F4, indicating the importance of major and school type in these measures.

**Table 5:** Results of Dunn’s Test With Bonferroni Correction for Significant Variables.


VARIABLE	COMPARISON	ROW MEAN-COL MEAN	*p*-VALUE

GPA by Major	Group 2 – Group 1	–0.47	1

GPA by Major	Group 3 – Group 1	–1.99	0.14

GPA by Major	Group 3 – Group 2	–1.59	0.34

GPA by Major	Group 4 – Group 1	–6.95	<0.0001***

GPA by Major	Group 4 – Group 2	–6.63	<0.0001***

GPA by Major	Group 4 – Group 3	–4.72	<0.0001***

F2 by Major	Group 2 – Group 1	–2.05	0.12

F2 by Major	Group 3 – Group 1	–1.57	0.35

F2 by Major	Group 3 – Group 2	0.33	1

F2 by Major	Group 4 – Group 1	0.55	1

F2 by Major	Group 4 – Group 2	2.48	0.04*

F2 by Major	Group 4 – Group 3	2.01	0.13

F4 by School	Group 2 – Group 1	–2.69	0.01**

F4 by School	Group 3 – Group 1	–2.23	0.04*

F4 by School	Group 3 – Group 2	0.65	0.77

Alexithymia by Major	Group 2 – Group 1	1.69	0.27

Alexithymia by Major	Group 3 – Group 1	1.94	0.16

Alexithymia by Major	Group 3 – Group 2	0.38	1

Alexithymia by Major	Group 4 – Group 1	3.21	0.004**

Alexithymia by Major	Group 4 – Group 2	1.69	0.27

Alexithymia by Major	Group 4 – Group 3	1.22	0.66

GPA by School	Group 2 – Group 1	–0.76	0.66

GPA by School	Group 3 – Group 1	–3.20	0.002**

GPA by School	Group 3 – Group 2	–1.70	0.13


***p < 0.001 – Very strong evidence of statistical significance. **p < 0.01 – Strong evidence of statistical significance. *p < 0.05 – Some evidence of statistical significance. *p* < 0.1 – No evidence of statistical significance.

### Causal Mediation Analysis

All school refusal behavior (SRB) factors did not have any significant moderation effects on the relationship between alexithymia and GPA (*p* > 0.05) (Table 6). The causal mediation analysis results across four models indicated that the ADE and TE of alexithymia (Alex) on GPA were significant in all models (*p*-value < 0.000). This suggests a strong direct negative relationship between alexithymia and GPA. However, the ACME, representing the indirect effect mediated through the factors F1, F2, F3, and F4, is non-significant in all models (*p*-value > 0.05). The proportion mediated (prop. mediated) was also non-significant, indicating that the mediators (F1, F2, F3, F4) did not explain a significant portion of the TE. These findings imply that Alexithymia’s impact on GPA was primarily direct rather than through these school refusal factors.

**Table 6:** Summary of Causal Mediation Analysis Results.


MODEL	ESTIMATE	CI LOWER	CI UPPER	*p*-VALUE

**F1 Mediator**				

ACME	0.0001	–0.007	0	0.98

ADE	–0.029	–0.039	–0.02	<.001***

TE	–0.029	–0.039	–0.02	<.001***

Prop. Med.	–0.0001	–0.003	0.02	0.98

**F2 Mediator**				

ACME	–0.001	–0.002	0	0.94

ADE	–0.029	–0.039	–0.02	<.001***

TE	–0.029	–0.039	–0.02	<.001***

Prop. Med.	0.017	–0.047	0.05	0.94

**F3 Mediator**				

ACME	–0.001	–0.002	0	0.27

ADE	–0.029	–0.038	–0.02	<.001***

TE	–0.029	–0.039	–0.02	<.001***

Prop. Med.	0.020	–0.018	0.09	0.27

**F4 Mediator**				

ACME	0.0002	–0.001	0	0.59

ADE	–0.030	–0.039	–0.02	<.001***

TE	–0.029	–0.039	–0.02	<.001***

Prop. Med.	–0.005	–0.045	0.02	0.59


***p < 0.001 – Very strong evidence of statistical significance. **p < 0.01 – Strong evidence of statistical significance. *p < 0.05 – Some evidence of statistical significance. *p* < 0.1 – No evidence of statistical significance.

### Hayes Mediation/Moderation Analysis

As Table 7 shows, the moderation analysis also demonstrated that alexithymia significantly affected GPA, negatively correlating with academic performance by a negative coefficient of 0.061 with a statistical significance of (*p* < 0.001) with the overall model, including academic majors, whereas having humanities major as the reference group as moderators, explaining approximately 32.27% of the variance in GPA (*R*^2^ = 0.3227). Specifically, while alexithymia consistently had a negative association with GPA across all majors, the extent of its effect size varied by field of study. The interactions between alexithymia and Major 2 (experimental sciences), Major 3 (mathematics), and Major 4 (other disciplines) were statistically significant, showing that the association of alexithymia and GPA were less for these groups than for students in the humanities (Major 1). The strongest moderation was observed in Major 4 (other diciplines), where the negative effect of alexithymia on GPA was considerably buffered (β = 0.0561, *p* < 0.001), indicating a significant interaction between individual psychological factors and the academic environment in this field.

**Table 7:** Moderating Effects of Academic Major on the Relationship Between Alexithymia and GPA.


INTERACTION	COEFFICIENT	SE	*t*-value	*p*-value	LOWER CI	UPPER CI

Alex × Experimental Sciences	0.035	0.01	2.90	0.004	0.011	0.059

Alex × Mathematics	0.050	0.01	3.67	<0.001	0.023	0.078

Alex × Other Disciplines	0.056	0.01	4.33	<0.0001	0.031	0.082


Further analysis using the PROCESS Macro in SPSS explored the moderating effects of school type on the association between alexithymia and GPA. The results indicated non-significant (*p* > 0.05) interactions between alexithymia and school types (public, private, gifted), suggesting that the negative association between alexithymia and GPA was consistent across educational settings (*R*^2^ = 0.1522), indicating that the model explains about 15.22% of the variance in GPA.

**Figure 3:**
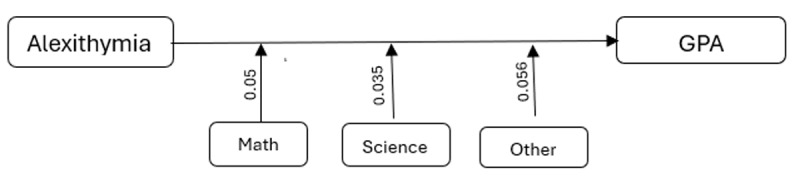
Moderating Effect Sizes of Majors on GPA.

Hayes analysis also revealed strong evidence that alexithymia statistically predicted GPA with β = –0.35 (*p* < 0.001) (Figure 3). For every one-unit increase in alexithymia score, GPA decreased by approximately 0.35 points, holding all predictors at their reference value (zero). The generalized *R*^2^ of 0.1227 suggests that approximately 12.27% of the variation in GPA rates was explained by alexithymia scores. Also, the direct effects of F1 (avoidance of stimuli provoking negative affectivity) and F2 (escape from aversive social and/or evaluative situations) on GPA were significant with β = 0.33 (*p* = 0.009) and β = –0.34 (*p* = 0.01), respectively.

### Path Model of Effective Factors in GPA

**Figure 4:**
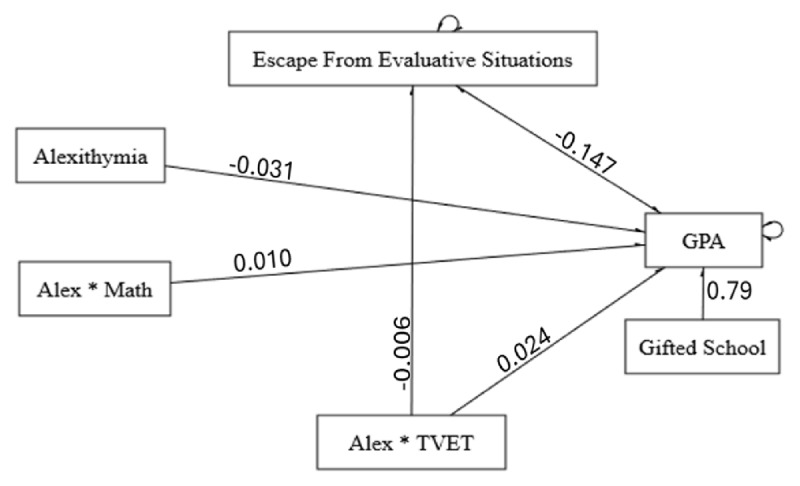
Path Model of Effective Factors on GPA.

The results from the path analysis, as shown in Figure 4, indicated an acceptable fit to the data, with a chi-square value of 1.499 and a *p*-value of 0.982, suggesting that the model fits the data exceptionally well, as Table 8 displays. Parameter estimates revealed significant relationships within the model: F2 was negatively affected by AlexOther (β = –0.006, *p* < 0.01). Alexithymia scores were negatively associated with the GPA (β = –0.031, *p* < 0.001), and positively associated with AlexOther (β = 0.025, *p* < 0.001), with gender and school type also showing significant relationships. Female students tended to get approximately 1.091 points higher average GPA than male students. These findings illustrate the complex interplay between psychological constructs and academic outcomes, demonstrating the utility of path analysis in understanding these relationships in educational settings.

**Table 8:** Parameter Estimates From the Path Model.


FIT INDEX	VALUE	RECOMMENDED THRESHOLD

Chi-square (*p*-value)	1.499 (0.982)	> 0.05 indicates a good fit

CFI	1	> 0.95 indicates a good fit

TLI	1.052	> 0.95 indicates a good fit

RMSEA	<0.0001	< 0.06 indicates a good fit

*p*-value RMSEA ≤ 0.050	0.998	–

SRMR	0.008	< 0.08 indicates a good fit


## Discussion

This cross-sectional study explored the relationship between alexithymia, school refusal behaviors, and GPA while considering differences in school type, academic major, and gender. School refusal behaviors include refusing to attend school and school withdrawal, which can affect students’ social and emotional development as well as their academic performance (Fremont, 2003; Havik & Ingul, 2021; Kearney & Diliberto, 2013; Kearney & Graczyk, 2020). Students engage in school refusal behaviors because of feeling anxious, stressed, or seeing the risk of violence and bullying (Dalsgaard et al., 2020; Kearney, 2007). However, school refusal behaviors have both short-term, poor academic performance and long-term effects as students who have severe school refusal behaviors are likely to have more mental health problems in adulthood (King et al., 2001). In addition, some causes of school refusal like anxiety and stress are also predictors of alexithymia, which is an emotional inability or trait that causes difficulty in recognizing and describing emotions (Berthoz et al., 1999; Farahani et al., 2022; Hong et al., 2024).

Our correlation analysis showed a strong negative relationship between alexithymia and GPA, which is consistent with the results of earlier studies (Romano et al., 2019). Alexithymia may negatively affect GPA and academic performance by increasing stress and anxiety, which impair emotional regulation and lead to academic burnout, characterized by cynicism and reduced motivation (Romano et al., 2019). We also found that attention-seeking behavior was positively associated with alexithymia, which could stem from externally oriented thinking (EOT), a key alexithymia trait, driving reliance on others to manage unarticulated distress (Preece et al., 2018).

Our Kruskal Walis and post-hoc tests showed that GPA differed between genders, school type, and academic major as females had higher GPAs, public school students achieved higher GPAs than gifted school students, and, lastly, students in humanities majors had the highest GPAs while those in Others, like technical and vocational education and training schools, had the lowest GPAs. The high performance of females may be due to higher achievement motivation and self-perceived academic achievement (Fischer et al., 2012). Public students getting higher GPAs requires further exploration because this study was conducted in 2023 and their GPAs were from the COVID-19 period. However, gifted schools have different curricula with different assessment criteria that might cause more difficulty in exams.

Previous studies have shown that mathematics, science, and humanities students’ poor mental health conditions, such those who refuse to go to schools to escape social interactions whether in class presentations or the school environment, which, in turn, negatively impacts their GPA (Larcombe et al., 2016; Stallman, 2010). In the current study, students in technical and vocational education and training courses had significant differences in their levels of F2 than students in experimental sciences, suggesting they felt more distress at school and, therefore, tended to refuse to attend to avoid social or evaluation situations. Also, we found significant differences in alexithymia levels among students studying technical and vocational education and training courses and those studying humanities, with the former having higher alexithymia levels. This may be due to their curriculum, which is more practice-oriented than course-based in experimental sciences (Phaeton & Stears, 2017).

Overall, students’ majors (mathematics and science) were significantly associated with their levels of alexithymia, which is in alignment with other studies showing majors have impacts on these disorders (Aleisa et al., 2022). For example, students in STEM fields exhibit higher levels of alexithymia due to the less emotive nature of the curriculum (Levant et al., 2009). However, in our study, there was no significant difference between alexithymia levels of students in mathematics, humanities, and experimental sciences.

Public students in our study showed the highest levels of F4, indicating that their school refusal was primarily motivated by the pursuit of tangible rewards outside of school. Here, not attending school for them is a positive reinforcement that provides access to enjoyable activities. This shows that the contextual factors (socioeconomic factors) of schools are also crucial in school refusal behaviors (Aleisa et al., 2022; Leduc et al., 2022).

Our causal mediation analysis showed school refusal behaviors did not mediate the relationship between alexithymia and GPA. However, the direct effects of F1 and F2 both showed significant associations with GPA, with F1 being positively related and F2 negatively related. It is logical to see that refusing school does not impact GPA as GPAs are a result of exam performance, not attendance, in Iran where most classes are lecture-based. However, one interesting finding in our study was that refusing school to avoid negative emotions such as stress and anxiety positively predicted GPA. This might be explained through the biopsychosocial model as refusing school would reduce stress and anxiety feelings so lower levels of cortisol (a biological factor) lead to enhanced short-term memory (Vedhara et al., 2000). Reduced anxiety itself is a psychological factor that leads to higher intrinsic motivation (Pei, 2023). Lastly, social factors behind why students with higher GPAs would refuse school attendance to avoid negative emotions include being in a supportive environment by family members or tutors who help them compensate for missed classes through targeted assistance or resources.

Our moderation analysis revealed no moderation effects of school type on the relationship between alexithymia and GPA, but academic majors had significant moderating effects on this relationship, especially studying technical and vocational education and training (TVET) courses had the largest moderation effect on this relationship. This suggests that studying this major is likely to strengthen the associations between alexithymia and GPA. TVET’s structured, hands-on focus minimizes emotional demands, allowing alexithymia students who struggle with identifying and expressing emotions to excel in tasks like machinery repair or coding, where precision trumps empathy.

Our path analysis showed similar results in how F2 was associated with GPA, but here F1 did not show any significant association and was removed from the model. In our path model, only the gifted school type had a significant association with GPA. Experimental science did not show any moderating effects on the relationship between alexithymia and GPA, revealing insights not seen in previous analyses. In our path model, school type (gifted school) and F2 (escaping from evaluative situations) showed the largest effect sizes among all predictors. Although our one-way Kruskal Walis and post-hoc tests showed higher GPAs among public students in our path model, only gifted schools showed a significant association with GPA. Findings from the Kruskal Walis test are likely due to its univariate focus on raw medians without controlling other predictors.

Our study investigated the relationship between alexithymia, school refusal behaviors, and GPA across school type, academic major, and gender, a framework that is well supported by the transactional model of stress and coping (Lazarus & Folkman, 1984). The model suggests that alexithymia, characterized by difficulty recognizing emotions (Berthoz et al., 1999) directly hampers GPA by impairing the ability to cope with stress, independent of school refusal behaviors, which, despite being driven by anxiety and stress (Kearney, 2007), did not mediate this link in our findings. Instead, school refusal reflects a separate response to stressors like bullying (Havik & Ingul, 2021), with short-term academic and long-term mental health impacts (King et al., 2001), whereas alexithymia’s effect on performance operates through uncoped emotional strain.

Implications of this study are that schools can prevent alexithymia and school refusal without screening tools like the PAQ by (a) embedding emotional literacy workshops into the curriculum, teaching students to recognize and express emotions through role-playing or journaling, thereby reducing stress-driven refusal (F1) and boosting motivation; (b) offering flexible attendance options, like hybrid learning, which can preempt avoidance of evaluative situations (F2) while maintaining GPA, especially for gifted or TVET students; and (c) fostering supportive environments with peer mentoring or teacher check-ins, which can counteract emotional isolation and distress, proactively addressing alexithymia’s roots and refusal triggers before they escalate, all without formal assessments. Also, our findings on school refusal behaviors underscore the need for targeted interventions tailored to school type and socioeconomic factors, particularly given the lack of mediation between alexithymia and GPA. In public schools, where students exhibited the highest levels of F4, pursuit of tangible rewards outside school, implementing mentorship programs, peer support initiatives, and structured extracurricular activities could redirect reward-seeking tendencies toward school engagement, countering socioeconomic drivers of refusal (Leduc et al., 2022).

Regarding the study’s limitation, we found that there is a need to use other complementary assessments to account better for the target population (Inglés et al., 2015). It has been suggested by previous international literature reviews that since SRAS-R shows a student’s present disposition of school refusal behavior, there is a need for assessments that can first predict the risk of this phenomenon for the future and account for the accuracy of SRAS-R (Inglés et al., 2015).

Another limitation of this study is its cross-sectional design, with data collected in 2023, which restricts our ability to infer causality or track changes over time; longitudinal studies could yield more robust and consistent findings than a single snapshot. Additionally, the data reflect a period when all students experienced online classes due to COVID-19, potentially influencing GPAs through altered learning environments, assessment methods, or reduced social pressures, which may not generalize to in-person settings.

## Conclusion

Our research findings indicate that alexithymia results in negative academic outcomes through increased stress and decreased motivation, but its outcomes vary depending on individual circumstances. The emotional intelligence theory (Salovey & Mayer, 1990) supports findings of the critical role of emotional awareness in mitigating the negative impact of alexithymia on academic performance. School refusal behaviors, notably F1 (avoiding negative emotions), can paradoxically enhance GPA by alleviating anxiety—supported by the biopsychosocial model. In contrast, F2 school refusal was negatively associated with GPA, according to a controlled path model analysis, which that identified gifted schools as a crucial factor for predicting GPAs. Academic programs focused on TVET strengthen the link between GPA and alexithymia, as these students, in particular, exhibit task-focused capabilities, yet public-school students tend to show higher grades. The research outcomes demonstrate that GPA loss from school refusal exists only in certain situations such as when students refuse to attend to escape for evaluative reasons but refusing attendance to avoid negative emotions relates to higher GPAs, and this warrants educational support strategies that take into account student emotional strengths in context.

## References

[1] Akram, A., & Arshad, T. (2022). Alexithymia reduction treatment: A pilot quasi-experimental study for remediation of alexithymia and its consequent effects on the general mental health of university students. Counselling and Psychotherapy Research, 22(4), 902–912. 10.1002/capr.12571

[2] Aleisa, M. A., Abdullah, N. S., Alqahtani, A. A. A., Aleisa, J. A. J., Algethami, M. R., & Alshahrani, N. Z. (2022). Association between alexithymia and depression among King Khalid University medical students: An analytical cross-sectional study. Healthcare, 10(9), 1703. 10.3390/healthcare10091703 36141315 PMC9498473

[3] Berthoz, S., Consoli, S., Perez-Diaz, F., & Jouvent, R. (1999). Alexithymia and anxiety: Compounded relationships? A psychometric study. European Psychiatry, 14(7), 372–378. 10.1016/s0924-9338(99)00233-3 10683621

[4] Dalsgaard, S., McGrath, J., Østergaard, S. D., Wray, N. R., Pedersen, C. B., Mortensen, P. B., & Petersen, L. (2020). Association of mental disorder in childhood and adolescence with subsequent educational achievement. JAMA Psychiatry, 77(8), 797. 10.1001/jamapsychiatry.2020.0217 32211833 PMC7097843

[5] Darvishi, A., Sanjari, E., & Shahraki, H. R. (2023). The prediction of alexithymia using depression, anxiety, stress, and demographics in undergraduate students. Journal of Biostatistics and Epidemiology, 8(4). 10.18502/jbe.v8i4.13356

[6] Ebrahimzadeh, K., Abadi, F. G. S., Anbi, S. A., & Abdolmohamadi, K. (2020). Psychometric properties of *School Refusal Assessment Scale-Revised*: Parent version in Iranian population. Practice in Clinical Psychology, 8(2), 123–132. 10.32598/jpcp.8.2.194.4

[7] Egger, H. L., Costello, J. E., & Angold, A. (2003). School refusal and psychiatric disorders: A community study. Journal of the American Academy of Child & Adolescent Psychiatry, 42(7), 797–807. 10.1097/01.chi.0000046865.56865.79 12819439

[8] Farahani, H., Azadfallah, P., Watson, P., Qaderi, K., Pasha, A., Dirmina, F., Esrafilian, F., Koulaie, B., Fayazi, N., Sepehrnia, N., Esfandiary, A., Abbasi, F. N., & Rashidi, K. (2022). Predicting the social-emotional competence based on childhood trauma, internalized shame, disability/shame scheme, cognitive flexibility, distress tolerance and alexithymia in an Iranian sample using Bayesian regression. Journal of Child & Adolescent Trauma, 16(2), 351–363. 10.1007/s40653-022-00501-1 37234828 PMC10205962

[9] Filippello, P., Buzzai, C., Costa, S., & Sorrenti, L. (2019a). School refusal and absenteeism: Perception of teacher behaviors, psychological basic needs, and academic achievement. Frontiers in Psychology, 10. 10.3389/fpsyg.2019.01471 PMC6610479 31316431

[10] Filippello, P., Buzzai, C., Messina, G., Mafodda, A. V., & Sorrenti, L. (2019b). School refusal in students with low academic performances and specific learning disorder. The role of self-esteem and perceived parental psychological control. International Journal of Disability Development and Education, 67(6), 592–607. 10.1080/1034912x.2019.1626006

[11] Fischer, F., Schult, J., & Hell, B. (2012). Sex differences in secondary school success: Why female students perform better. European Journal of Psychology of Education, 28(2), 529–543. 10.1007/s10212-012-0127-4

[12] Fremont, W. P. (2003). School refusal in children and adolescents. PubMed, 68(8), 1555–1560. https://pubmed.ncbi.nlm.nih.gov/14596443 14596443

[13] Gao, S., Mokhtarian, P. L., & Johnston, R. A. (2008). Nonnormality of data in structural equation models. Transportation Research Record Journal of the Transportation Research Board, 2082(1), 116–124. 10.3141/2082-14

[14] Hamaideh, S. (2016). Mental health nurses’ perceptions of patient safety culture in psychiatric settings. International Nursing Review, 64(4), 476–485. 10.1111/inr.12345 27966218

[15] Havik, T., Bru, E., & Ertesvåg, S. K. (2015). School factors associated with school refusal- and truancy-related reasons for school non-attendance. Social Psychology of Education, 18(2), 221–240. 10.1007/s11218-015-9293-y

[16] Havik, T., & Ingul, J. M. (2021). How to understand school refusal. Frontiers in Education, 6. 10.3389/feduc.2021.715177

[17] Heyne, D., Gren-Landell, M., Melvin, G., & Gentle-Genitty, C. (2018). Differentiation between school attendance problems: Why and how? Cognitive and Behavioral Practice, 26(1), 8–34. 10.1016/j.cbpra.2018.03.006

[18] Hong, Y., Chen, S., Chang, Y., Chang, C., & Chiang, H. (2024). The role of alexithymia in suicide ideation among Taiwanese army military personnel: A serial mediation model investigating the effects of perceived stress and depression. Stress and Health, 40(5). 10.1002/smi.3405 38660797

[19] Imai, K., Keele, L., Tingley, D., & Yamamoto, T. (2009). Causal mediation analysis using R. In H. Vinod (Ed.), Advances in social science research using R. Lecture notes in statistics (vol. 196, pp. 129–154). Springer. 10.1007/978-1-4419-1764-5_8

[20] Inglés, C. J., Gonzálvez, C., García-Fernández, J. M., Vicent, M., & Martínez-Monteagudo, M. C. (2015). Current status of research on school refusal. European Journal of Education and Psychology, 8(1), 37–52. 10.30552/ejep.v8i1.135

[21] Iuso, S., Severo, M., Ventriglio, A., Bellomo, A., & Limone, P. (2022). Psychoeducation reduces Alexithymia and modulates anger expression in a school setting. Children, 9(9), 1418. 10.3390/children9091418 36138728 PMC9498159

[22] Kearney, C. (2007). School absenteeism and school refusal behavior in youth: A contemporary review. Clinical Psychology Review, 28(3), 451–471. 10.1016/j.cpr.2007.07.012 17720288

[23] Kearney, C. A., & Diliberto, R. (2013). School refusal behavior. In S. G. Hofmann (Ed.), The Wiley handbook of cognitive behavioral therapy (pp. 875–892). Wiley. 10.1002/9781118528563.wbcbt37

[24] Kearney, C. A., & Graczyk, P. A. (2020). A multi-dimensional, multi-tiered system of support model to promote school attendance and address school absenteeism. Clinical Child and Family Psychology Review, 23(3), 316–337. 10.1007/s10567-020-00317-1 32274598

[25] Kearney, C. A., Turner, D., & Gauger, M. (2010). School refusal behavior. In W. E. Craighead & C. B. Nemeroff (Eds.), The Corsini encyclopedia of psychology and behavioral science, Vols. 1–2. Wiley. 10.1002/9780470479216.corpsy0827

[26] King, N. J., Heyne, D., Tonge, B., Gullone, E., & Ollendick, T. H. (2001). School refusal: Categorical diagnoses, functional analysis and treatment planning. Clinical Psychology & Psychotherapy, 8(5), 352–360. 10.1002/cpp.313

[27] Larcombe, W., Finch, S., Sore, R., Murray, C. M., Kentish, S., Mulder, R. A., … & Williams, D. A. (2016). Prevalence and socio-demographic correlates of psychological distress among students at an Australian university. Studies in Higher Education, 41(6), 1074–1091. 10.1080/03075079.2014.966072

[28] Lashkari, A., Dehghani, M., Sadeghi-Firoozabadi, V., Heidari, M., & Khatibi, A. (2021). Further support for the psychometric properties of the Farsi version of Perth Alexithymia questionnaire. Frontiers in Psychology, 12. 10.3389/fpsyg.2021.657660 PMC8079730 33935916

[29] Lazarus, R. S., & Folkman, S. (1984). Stress, appraisal, and coping. Springer Publishing Company.

[30] Leduc, K., Tougas, A., Robert, V., & Boulanger, C. (2022). School refusal in youth: A systematic review of ecological factors. Child Psychiatry & Human Development, 55(4), 1044–1062. 10.1007/s10578-022-01469-7 36422762 PMC9686247

[31] Levant, R. F., Hall, R. J., Williams, C. M., & Hasan, N. T. (2009). Gender differences in alexithymia. Psychology of Men & Masculinity, 10(3), 190–203. 10.1037/a0015652

[32] MacFarland, T. W., & Yates, J. M. (2016). Kruskal–Wallis H-Test for one-way analysis of variance (ANOVA) by ranks. In T. W. MacFarland & J. M. Yates (Eds.), Introduction to nonparametric statistics for the biological sciences using R (pp. 177–211). Springer. 10.1007/978-3-319-30634-6_6

[33] Martin, A. J., & Marsh, H. W. (2006). Academic resilience and its psychological and educational correlates: A construct validity approach. Psychology in the Schools, 43(3), 267–281. 10.1002/pits.20149

[34] Mcshane, G., Walter, G., & Rey, J. M. (2001). Characteristics of adolescents with school refusal. Australian & New Zealand Journal of Psychiatry, 35(6), 822–826. 10.1046/j.1440-1614.2001.00955.x 11990893

[35] Pei, J. (2023). Exploring the relationships between motivation and anxiety of college students English language learning. Lecture Notes in Education Psychology and Public Media, 31(1), 59–64. 10.54254/2753-7048/31/20231821

[36] Pfeffer, J., & Salancik, G. R. (1978). The external control of organizations: A resource dependence perspective. Harper & Row.

[37] Phaeton, M. J., & Stears, M. (2017). Exploring the alignment of the intended and implemented curriculum through teachers’ interpretation: A case study of A-level biology practical work. Eurasia Journal of Mathematics, Science and Technology Education, 13(3), 723–740. 10.12973/eurasia.2017.00640a

[38] Preece, D., Becerra, R., Robinson, K., Dandy, J., & Allan, A. (2018). Perth Alexithymia Questionnaire [Dataset]. APA PsycTests. 10.1037/t68335-000

[39] Richardson, M., Abraham, C., & Bond, R. (2012). Psychological correlates of university students’ academic performance: A systematic review and meta-analysis. Psychological Bulletin, 138(2), 353–387. 10.1037/a0026838 22352812

[40] Romano, L., Buonomo, I., Callea, A., & Fiorilli, C. (2019). Alexithymia in young people’s academic career: The mediating role of anxiety and resilience. The Journal of Genetic Psychology, 180(4–5), 157–169. 10.1080/00221325.2019.1620675 31165680

[41] Salovey, P., & Mayer, J. D. (1990). Emotional intelligence. Imagination, Cognition, and Personality, 9(3), 185–211. 10.2190/DUGG-P24E-52WK-6CDG

[42] Sifneos, P. (1973). The prevalence of “alexithymic” characteristics in psychosomatic patients. Psychotherapy and Psychosomatics, 22(2–6), 255–262. 10.1159/000286529 4770536

[43] Stallman, H. M. (2010). Psychological distress in university students: A comparison with general population data. Australian Psychologist, 45(4), 286–294. 10.1080/00050067.2010.482109

[44] Swansea, K. R. (2010). Finding strategic solutions to reduce truancy. Research in Education, 84(1), 1–18. 10.7227/rie.84.1

[45] Valkenburg, P. M., & Peter, J. (2013). The differential susceptibility to media effects model. Journal of Communication, 63(2), 221–243. 10.1111/jcom.12024

[46] Vedhara, K., Hyde, J., Gilchrist, I., Tytherleigh, M., & Plummer, S. (2000). Acute stress, memory, attention and cortisol. Psychoneuroendocrinology, 25(6), 535–549. 10.1016/s0306-4530(00)00008-1 10840167

[47] Westrick, P. A., Le, H., Robbins, S. B., Radunzel, J. M. R., & Schmidt, F. L. (2015). College performance and retention: A meta-analysis of the predictive validities of ACT^®^ scores, high school grades, and SES. Educational Assessment, 20(1), 23–45. 10.1080/10627197.2015.997614

[48] Willingham, D. T. (2009). Why don’t students like school?: A cognitive scientist answers questions about how the mind works and what it means for the classroom. http://hacend.is-best.net/h/why-dont-students-like-school-a-cognitive-scientist-answers-questions-about-how-the-mind-works-and-what-it-means-for-the-classroom-by-daniel-t-willingham.pdf

[49] Wilmot, A., Hasking, P., Leitão, S., Hill, E., & Boyes, M. (2023). Understanding mental health in developmental dyslexia: A scoping review. International Journal of Environmental Research and Public Health, 20(2), 1653. 10.3390/ijerph20021653 36674408 PMC9864451

